# Supplementary material for: scPNMF: sparse gene encoding of single cells to facilitate gene selection for targeted gene profiling
Source: Bioinformatics. 2021 Jul 12;37(Suppl 1):i358–66. doi: 10.1093/bioinformatics/btab273 (PMC8275345; doi:10.1093/bioinformatics/btab273)
Supplement: btab273_Supplementary_Data [file btab273_supplementary_data.pdf]

Supplementary Information

scPNMF: sparse gene encoding of single cells to facilitate gene selection for targeted gene profiling

Dongyuan Song, Kexin Li, Zachary Hemminger, Roy Wollman, and Jingyi Jessica Li

S1 Choice of parameters and robustness analysis

S1.1 Low rank K

In the development of scPNMF, motivated by the objective function of the PNMF method,

$$\min_{\mathbf{W}\in\mathbb{R}_{\geq 0}^{p\times K}} \|\mathbf{X}-\mathbf{W}\mathbf{W}^{\mathsf{T}}\mathbf{X}\|\,, \tag{S1}$$

PNMF aims to inherit the advantages of PCA such as the basis orthogonality and the ability to project new data. However, a key constraint in PCA, $\mathbf{W}^{\mathsf{T}}\mathbf{W}=\mathbf{I}$, is relaxed to satisfy the constraint $\mathbf{W}\geq 0$ in PNMF. To make PNMF closer to PCA and thus approximately achieve these two nice properties, we propose to use the normalized difference between $\mathbf{W}^{\mathsf{T}}\mathbf{W}$ and $\mathbf{I}$ to measure the orthonality of $\mathbf{W}$:

$$dev.ortho = \|\mathbf{I}-\mathbf{W}^{\mathsf{T}}\mathbf{W}\|/K^2\,. \tag{S2}$$

It naturally gives rise to a method to determine the number of bases, K: first perform PNMF for a sequence of K's; second, for each K, we calculate the *dev.ortho* measure for the corresponding $\mathbf{W}\in\mathbb{R}_{\geq 0}^{p\times K}$; third, we plot *dev.ortho* against K. Users can decide K when *dev.ortho* reaches stability or there is a clear elbow in the graph.

In Fig. S1, using the Zheng4 [Duò et al., 2018] dataset, we demonstrate that (1) the *dev.ortho* measure is highly correlated with the performance of $\mathbf{W}$ in the downstream analysis; (2) in real data application, the *dev.ortho* measure shows a clear elbow pattern, which can help users determine K.

Empirically, we see that *dev.ortho* reaches stability at $K = 20$ for most scRNA-seq data. For the purpose of guiding users and saving computational time, we set the default number of bases in scPNMF to be $K = 20$.

S1.2 R_0: threshold for correlations between score vectors and cell library sizes in "scPNMF step II: basis selection"

In real data application, the threshold for correlations between score vectors and cell library sizes in "scPNMF step II: basis selection," R_0, needs to be pre-defined. We consider thresholds with one decimal digit resolution $\{0.5, 0.6, 0.7, 0.8, 0.9\}$ because of the convention in the field. By running the K-means clustering on the seven datasets (see Table S4) and applying these threshold, as shown in Fig. S2, we suggest setting $R_0 = 0.7$ for $K \geq 10$, and more conservatively, $R_0 = 0.8$ when the basis number K is small ($K < 10$).

S2 Functional annotation

We use the R package `clusterProfiler` [Yu et al., 2012] to perform the GO analysis. We set the gene ontology as "biological processes (BP)" and the adjusted p-value cutoff as 0.1. The output GO terms are simplified by `clusterProfiler`.

In this paper, we only perform a very conservative filtering based on functionality. We define the common housekeeping gene list to include *ACTB*, *ACTG1*, *B2M*, *GAPDH*, and *MALAT1*. If a basis' top 10 highly weighted genes contain any of these five genes, this basis will be filtered out.

S3 Data preprocessing

scPNMF only performs minimum data preprocessing to avoid information loss. Denote a scRNA-seq count matrix as $\mathbf{X}^C \in \mathbb{N}^{p\times n}$, with rows representing p genes and columns representing n cells. scPNMF creates the log count matrix $\mathbf{X} \in \mathbb{R}_{\geq 0}^{p\times n}$ by taking the log transformation of $\mathbf{X}^C$ with a pseudo count of 1:

$$\mathbf{X}_{ij} = \log\left(\mathbf{X}_{ij}^C + 1\right), \qquad i = 1, \ldots, p;\ j = 1, \ldots, n. \tag{S3}$$

scPNMF takes the log count matrix $\mathbf{X} \in \mathbb{R}_{\geq 0}^{p\times n}$ as the input. With the log transformation, the effect of a few extremely large counts will be alleviated, and the transformed values will have more Gaussian-like distributions, an assumption assumed by many methods. We introduce the pseudo count of 1 to avoid negative infinite values in the later PNMF optimization step.

For the scRNA-seq data used in this paper (Table S2), we filter out the genes that are expressed in fewer than 5% of the cells, and then we filter out the cells that are expressed in fewer than 5% of the remaining genes. Additionally, *MALAT1*, mitochondrial genes, and ribosomal genes are removed from two datasets, PBMC10x and PBMCSmartSeq, according to the reference paper [Ding et al., 2020]. Users may customize the filtering process before they input the log count matrix $\mathbf{X}$ into scPNMF.

S4 Details about informative gene selection and cell clustering

In this paper, we compare scPNMF with 11 other informative gene selection methods (Table S3). Some gene selection methods cannot let users pre-define an arbitrary gene number; for such methods (e.g., SCMarker [Wang et al., 2019]), we adjust their tuning parameters until their output gene numbers approximately equal the desired gene number.

We apply three clustering algorithms, Louvain clustering (by Seurat), K-means clustering (by R function `kmeans`), and hierarchical clustering (by R function `hclust`). We perform PCA on informative genes and use the top 20 PCs for cell clustering. We use $U = \{u_1, \ldots, u_P\}$ to denote the true partition of P classes and $V = \{v_1, \ldots, v_K\}$ to denote the partition given by clustering results. Let n_i and n_j be the numbers of observations in class u_i and cluster v_j respectively, and n_{ij} denotes the number of observations in both class u_i and cluster v_j. The adjusted Rand index (ARI) is calculated as

$$\frac{\sum_{i=1}^P \sum_{j=1}^K \binom{n_{ij}}{2} - \left[\sum_{i=1}^P \binom{n_{i\cdot}}{2} \sum_{j=1}^K \binom{n_{\cdot j}}{2}\right] / \binom{n}{2}}{\frac{1}{2}\left[\sum_{i=1}^P \binom{n_{i\cdot}}{2} + \sum_{j=1}^K \binom{n_{\cdot j}}{2}\right] - \left[\sum_{i=1}^P \binom{n_{i\cdot}}{2} \sum_{j=1}^K \binom{n_{\cdot j}}{2}\right] / \binom{n}{2}} \tag{S4}$$

where $n = \sum_{i=1}^P n_{i\cdot} = \sum_{j=1}^K n_{\cdot j}$. An ARI value close to 1 means more accurately inferred clusters. Regarding the choice of tuning parameter values (the resolution parameter r in Louvain clustering and the number of clusters k in K-means and hierarchical clustering), we consider the following parameter values:

$$r \in \{0.02, 0.04, 0.06, 0.08, 0.1, 0.2, 0.3, 0.4, 0.5, 0.6, 0.7, 0.8, 0.9, 1.0\}, \quad k \in \{2, 3, 4, \cdots, 15\}, \tag{S5}$$

and we use the average of the top three high ARI values (across the parameter combinations) as the final output.

S5 Details about new data projection and cell type prediction

We use two datasets, Zheng8 and PBMC10x, as the reference scRNA-seq datasets. For the Zheng8 dataset, we first use scDesign2 [Sun et al., 2020] to learn the underlying parameters, and then we simulate a new dataset with the same genes and cell types but a 100-time larger sequencing depth compared to the Zheng8 dataset. For the PBMC10x dataset, we use the PBMCSmartSeq dataset, which measures the exact same example by Smart-seq2 and contains all genes measured in PBMC10x. Given M selected genes, the simulated

Zheng8 and PBMC10x are pruned to contain only those genes, and the pruned datasets serve as the "pseudo" targeted gene profiling datasets that only have the M genes measured.

For cell type prediction, we project every targeted gene profiling dataset and its scRNA-seq reference onto the same low-dimensional space, which mainly follows the idea of scPred [Alquicira-Hernandez et al., 2019]. When applying scPNMF, we use the weight matrix $\mathbf{W}_{S,(M)}$ to project both the reference dataset and the targeted gene profiling dataset. For other gene selection methods, we first subset the reference dataset with only M selected genes, run PCA to obtain a weight matrix $\mathbf{W}_{\mathrm{PCA}}$, and then use it to project both the reference dataset and targeted gene profiling dataset, both containing only M genes. After obtaining the two sets of low-dimensional embeddings of reference and targeted gene profiling datasets, we run the Harmony algorithm [Korsunsky et al., 2019] to remove the technical variations between these two sets of low-dimensional embeddings. Then we apply three classification algorithms, random forest (`rf`), k-nearest neighbors (`knn`), and support vector machine with radial kernel (`svmRadial`) in the R package `caret` [Kuhn et al., 2008], for cell type prediction. The tuning parameters are selected by 5-fold cross-validation with three repeats.

S6 Data normalization by cell library size

S6.1 Why scPNMF does not use normalized data as input

By default, scPNMF takes the raw data without normalization (e.g., regressing out the cell library size [Hafemeister and Satija, 2019]). In practice, scPNMF can be applied to such pre-processed scRNA-seq data, and then it does not need to remove the factors correlated with cell library size in its basis selection step. However, we have two reasons to prefer the default procedure.

1. Normalizing by cell library size is inappropriate for targeted gene profiling. In scRNA-seq, the cell library size is the total count in a cell. However, in targeted gene profiling, the cell sequencing depth can not be accurately estimated since only a small subset of genes is captured. For instance, `Seurat` claims that in the analysis of spatial data (a type of targeted gene profiling data), "force each data point to have the same underlying 'size' after normalization, can be problematic" [Seurat, 2021]. To make sure that the genes selected based on scRNA-seq are informative for designing targeted gene profiling experiments, we prefer to use raw data without normalizing cell library sizes.

2. Cell library size can be informative for distinguishing cell types. Some studies have observed that cell library sizes are significantly different between some cell types and thus serve as a useful feature for distinguishing them [Monaco et al., 2019, Kim et al., 2020], a phenomenon we have also encountered in our data analysis. Therefore, normalization by cell library size is not always desirable. scPNMF avoids this issue by using unnormalized data, and if a factor is correlated with cell library size but also shows a significant multimodal pattern, the factor will be preserved for downstream analysis.

S6.2 Normalization on the score matrix output by scPNMF

If users want to directly remove the effects of cell library size, they may choose the option of "regressing out cell library size by cell type" in the `scPNMF` package. The reason why we do not set it as the default is that scPNMF is designed as an unsupervised method **without cell type information**. Therefore, if cell type labels are not provided, it is impossible to regress out cell library size in a cell-type-specific manner. To overcome this no-cell-type-label issue, scPNMF applies a new clustering algorithm, "K-lines clustering" [Li et al., 2018], to identify more than one linear relationship, if existent, between a basis and cell library size. Below we describe our algorithm.

In the *score matrix* $\mathbf{S} = \mathbf{W}^{\mathsf{T}}\mathbf{X} \in \mathbb{R}_{\geq 0}^{K \times n}$, whose K rows correspond to bases and whose n columns represent cells, the k-th row of $\mathbf{S}$, denoted by $\boldsymbol{s}_k^{\mathsf{T}}$, contains the *scores* (i.e., coordinates) of n cells in the k-th basis. For $\boldsymbol{s}_k^{\mathsf{T}}$, we assume that it is composed of cell groups $C_1, \ldots, C_{N_k}$, which correspond to either pre-defined cell types or clusters obtained by K-lines clustering. That is, $C_1 \cup \cdots \cup C_{N_k} = \{1, \ldots, n\}$. Therefore, for group C_r, $r = 1, \ldots, N_k$, we fit a linear model:

$$s_{ik} = \beta_{0k}^{(r)} + \beta_{1k}^{(r)} d_i + \epsilon_i, \quad i \in C_r \, ,$$

where d_i is cell i's library size, and obtains estimates $\hat{\beta}_{0k}^{(r)}$ and $\hat{\beta}_{1k}^{(r)}$, as well as residuals e_i, $i \in C_r$. Then, we define the "corrected" score of cell i in group C_r as

$$u_{ik} = \hat{\beta}_{0k}^{(r)} + \hat{\beta}_{1k}^{(r)} \bar{d}^{(r)} + e_i, \quad i \in C_r \, ,$$

where $\bar{d}^{(r)}$ is the mean cell library size in group C_r. The corrected score matrix $\mathbf{U}$ is used for downstream analysis such as dimensionality reduction. We observe that, using the corrected scores, the "stretching" shape within each cell type is removed, and cell types are better distinguished in UMAP visualization (Fig. S3).

Although this correction is useful, we argue that it should be used with caution since the results depend on cell type/cluster labels. In an unsupervised setting, we recommend users to follow the basis selection criteria as we described in our paper.

S7 Comparison between PNMF and NMF

PNMF, the first step of `scPNMF`, outputs a much more sparse representation of a scRNA-seq dataset than NMF does. Using the FregGold dataset [Freytag et al., 2018] and $K = 5$ bases, we demonstrate that the weight matrix of PNMF is highly sparse (42.6% zeros) and has largely mutually exclusive bases, while the zero proportion in the weight matrix of NMF is only 1.1%, and the bases are much less mutually exclusive (Fig. S4). These results suggest that PNMF bases are concentrated on a small set of genes and correspond to gene groups with distinct functions. Moreover, we demonstrate that, when applied to the seven scRNA-seq datasets (Table S4), `scPNMF` outperforms its variant that replaces PNMF by NMF in the first step (Fig. S5)

`scPNMF` also has the functionality of outputting a projection matrix $\mathbf{W}$ that can project new cells onto the latent space, which `scPNMF` learns from reference cells. This functionality enables the alignment of new data with the reference data in the same low-dimensional space and facilitates cell type prediction in the new data. In contrast, NMF does not output a projection matrix, and the basis-by-cell matrix it outputs does not satisfy the requirement of a projection matrix.

The reason why we cannot simply select genes from the NMF weight matrix and use these genes to align new data with reference data is that the selected gene number (usually in hundreds) would be much greater than the number of bases in `scPNMF`'s projection matrix. Otherwise, if the selected gene number is too small, we would lose biological information for aligning cells, not to mention that it is not straightforward to select a small number of genes from a not-so-sparse NMF weight matrix. Due to the well known curse of dimensionality, we deem it reasonable to use a low-dimensional space, instead of hundreds of genes, to align cells. `scPNMF` essentially combines gene selection and dimensionality reduction into one step by directly providing the projection matrix, and we demonstrate that `scPNMF` has good performance in applications.

S8 Comparison with f-scLVM

The factorial single-cell latent variable model (f-scLVM) is a Bayesian method based on factor analysis that can jointly refine gene set annotations and infer factors without annotation. Similar to our scPNMF, f-scLVM indeed can also learn sparse and interpretable factors [Buettner et al., 2017]. However, scPNMF differs from f-scLVM in its required input data, main goal, and model construction. As a result, scPNMF has better performance in informative gene selection for targeted gene profiling, which is its major goal. Moreover, we find scPNMF more computationally efficient than f-scLVM.

S8.1 Differences in input data

While scPNMF only requires a gene-by-cell count matrix as input, f-scLVM additionally requires pre-defined gene sets for its model fitting.

S8.2 Differences in main goal and model

scPNMF aims to select a limited number of informative genes for targeted gene profiling based on existing scRNA-seq data. It finds the set of informative genes by learning a low-dimensional embedding of cells so

that the bases correspond to sparse and mutually exclusive gene groups, and further selecting bases based on functional annotations (optional), correlation screening, and multimodality testing to remove uninformative bases that cannot distinguish cell types.

In contrast, f-scLVM focuses more on decomposing scRNA-seq datasets into interpretable components. It jointly infers both annotated and unannotated factors, including confounders, and refines the pre-defined gene sets in a data driven manner. The model can be written as:

$$\mathbf{Y} = \underbrace{\sum_{c=1}^{C} \mathbf{u}_c \mathbf{V}_c^\mathsf{T}}_{\text{cell covariates}} + \underbrace{\sum_{a=1}^{A} \mathbf{p}_a \mathbf{R}_a^\mathsf{T}}_{\text{annotated factors}} + \underbrace{\sum_{h=1}^{H} \mathbf{s}_h \mathbf{Q}_h^\mathsf{T}}_{\text{unannotated factors}} + \mathbf{\Psi} \tag{S6}$$

$$= \mathbf{X}\mathbf{W}^\mathsf{T} + \mathbf{\Psi}. \tag{S7}$$

Here, $\mathbf{Y}$ denotes the cell-by-gene gene expression matrix; the vectors $\mathbf{u}_c$, $\mathbf{p}_a$, $\mathbf{s}_h$ correspond to known cell covariates, as well as cell states for annotated and unannotated factors; and $\mathbf{V}_c$, $\mathbf{R}_a$, $\mathbf{Q}_h$ are the corresponding regulatory weights of a given factor on all genes; the matrix $\mathbf{\Psi}$ denotes residual noise. We then collapse the vectors of factors and weights into activation matrices $\mathbf{X} = [\mathbf{u}_1, \ldots, \mathbf{u}_C, \mathbf{p}_1, \ldots, \mathbf{p}_A, \mathbf{s}_1, \ldots, \mathbf{s}_H]$ and $\mathbf{W} = [\mathbf{V}_1, \ldots, \mathbf{V}_C, \mathbf{R}_1, \ldots, \mathbf{R}_A, \mathbf{Q}_1, \ldots, \mathbf{Q}_H]$.

f-scLVM is not originally designed for selecting informative genes from the gene expression matrix alone. Although the f-scLVM authors have discussed about identifying an augmented gene set specific to each factor, the identified genes are for interpreting the factors but not for capturing the overall biological variations of cells or distinguishing cell types.

S8.3 Results for informative gene selection

Although there is no description about informative gene selection in the f-scLVM paper, inspired by the bilinear model structure (eq. (S7)), we have used f-scLVM in two ways to select n informative genes from its estimated weight matrix $\mathbf{W}$, whose columns are factors' loading vectors and rows are genes. Note that $\mathbf{W}$ is not a direct output of the f-scLVM software package.

- Across-factor: select informative genes based on their maximum loadings across factors (i.e., take the maximum of each row of $\mathbf{W}$; then pick the n genes with the largest row maxima).
- Per-factor: select top $\sim \lceil n/K \rceil$ informative genes for each factor, where K is the number of factors (i.e., pick the $\sim \lceil n/K \rceil$ genes with the largest loadings in each column of $\mathbf{W}$); then take union of the K informative gene sets. Note that the union may contain fewer than n genes due to the possible overlaps of gene sets.

The f-scLVM results are based on R package `slalom` (version 1.10.0) and default parameter values (Gene set annotations: the MSigDB core processes database (hallmark gene sets H) v7.2; number of hidden factors: 5; minimum number of genes to retain a gene set: 10). Similar as in Section 3.3, we comprehensively benchmark scPNMF and f-scLVM on seven scRNA-seq datasets (Table S4) using three clustering methods (Louvain clustering, K-means clustering, and hierarchical clustering). Using the adjusted Rank index (ARI) as the metric of clustering accuracy, we calculate the ARI values of the three clustering methods on each dataset using 20, 50, 100, 200, 500 selected informative genes, which are the commonly used gene numbers in targeted gene profiling.

Fig. S6 shows that scPNMF consistently has the highest overall ARI values across datasets and clustering methods. scPNMF leads to more stable overall average ARI values under varying numbers of informative genes, indicating its stronger robustness to the gene number constraint of targeted gene profiling. It is worth noting that scPNMF works well even when the number of informative genes is as small as 20.

Similar as in Section 3.3, Fig. S7 shows the UMAP visualization of cells in the Zheng4 dataset based on the 100 informative genes selected by scPNMF and f-scLVM. scPNMF leads to a clear separation of naive cytotoxic T cells and regulatory T cells, while f-scLVM-Across-factor and f-scLVM-Per-factor cannot, even though f-scLVM-Per-factor incorporates slightly more (123) informative genes.

S8.4 Computational time

Table S1: Running time of scPNMF and f-scLVM in minutes

Dataset	scPNMF Running Time (mins)	f-scLVM Running Time (mins)
Darmanis	27.95	178.57
FreytagGold	38.87	474.61
Tirosh	23.47	1172.91
PBMC10x	22.87	733.36
PBMCSmartSeq	48.88	107.92
Zheng4	0.90	53.89
Zheng8	1.12	111.89

scPNMF is more time-efficient than f-scLVM across diverse scRNA-seq datasets. We have run both software packages on a PC with 3.4 GHz Quad-Core Intel Core i5 and 8GB RAM. The f-scLVM results are based on R package `slalom` (version 1.10.0) and default parameter values. In Table S1, we can see that scPNMF runs 2.2x $\sim$ 100x faster than f-scLVM.

Table S2: Top 10 high weight genes in each PNMF basis of the FretagGold dataset

Basis	Gene symbol	Description
1	*RPS2, TMSB4X, GAPDH, RPL41, RPL13, FTH1, MALAT1, COX2, RPL10, RPS18*	Highly expressed housekeeping genes
2	*CD74, PTGR1, HLA-B, ALDH3A1, C15orf48, LCN2, IGFBP3, SAA1, CXCL1, HLA-DRA*	Immune-related genes
3	*SEC61G, CDK4, CCN1, G0S2, ELOC, VOPP1, EGFR, F3, CDKN2A, EPCAM*	Tumor-related genes (oncogenes, tumor suppressor genes)
4	*H4C3, CKS1B, HMGB2, SMC4, PTTG1, KPNA2, CCNB1, CDKN3, CKS2, CDC20*	Genes related to mitotic cell cycle
5	*HSPB1, UBE2S, CALD1, TMEM256, FIS1, ISOC2, ZNHIT1, C20orf27, NDUFA3, PPP2R1A*	Genes related to mitochondrion

Table S3: Overview of informative gene selection methods used in this study

Method	User-defined gene #	Language	Package	Reference
corFS	Yes	R	`M3Drop` (version 1.14.0)	[Andrews and Hemberg, 2019]
DANB	Yes	R	`M3Drop` (version 1.14.0)	[Andrews and Hemberg, 2019]
GiniClust	Yes	R	`M3Drop` (version 1.14.0)	[Andrews and Hemberg, 2019]
irlbaPcaFS	Yes	R	`M3Drop` (version 1.14.0)	[Andrews and Hemberg, 2019]
M3Drop	Yes	R	`M3Drop` (version 1.14.0)	[Andrews and Hemberg, 2019, Jiang et al., 2016]
Scanpy	Yes	Python	`Scanpy` (version 1.6.0)	[Wolf et al., 2018]
SCMarker	No	R	`SCMarker`[1]	[Wang et al., 2019]
scran	Yes	R	`scran` (version 1.18.3)	[Lun et al., 2016]
SeuratDISP	Yes	R	`Seurat` (version 3.2.2)	[Stuart et al., 2019, Hafemeister and Satija, 2019]
SeuratMVP	No	R	`Seurat` (version 3.2.2)	[Stuart et al., 2019]
SeuratVST	Yes	R	`Seurat` (version 3.2.2)	[Stuart et al., 2019]
f-scLVM	No	R	`slalom` (version 1.10.0)	[Buettner et al., 2017]

1: Due to failure in `SCMarker` R package installation, we run the R script downloaded from https://github.com/KChen-lab/SCMarker on September 17, 2020.

Table S4: Overview of datasets used in this study

Dataset	Sequencing protocol	Gene #	Cell #	Cell type #	True label	Description	Ref
Darmanis	Smart-Seq2	13256	420	8	No	Human adult cortical samples	[Darmanis et al., 2015]
FreytagGold	10xGenomics Chromium	15410	925	3	Yes	Mixture of human lung adenocarcinoma cell lines	[Freytag et al., 2018]
Tirosh	Smart-Seq2	11934	2887	6	No	Human melanoma tumors	[Tirosh et al., 2016]
PBMC10x	10xGenomics Chromium	11714	3308	9	No	Human peripheral blood mononuclear cells. 10x-v2 for sample 1 in the original paper.	[Ding et al., 2020]
PBMCSmartSeq	Smart-Seq2	17479	273	6	No	Human peripheral blood mononuclear cells. Smart-Seq2 for sample 1 in the original paper.	[Ding et al., 2020]
Zheng4	10xGenomics GemCode	2192	3994	4	Yes	Mixture of human peripheral blood mononuclear cells	[Duò et al., 2018, Zheng et al., 2017]
Zheng8	10xGenomics GemCode	2390	3994	8	Yes	Mixture of human peripheral blood mononuclear cells	[Duò et al., 2018, Zheng et al., 2017]

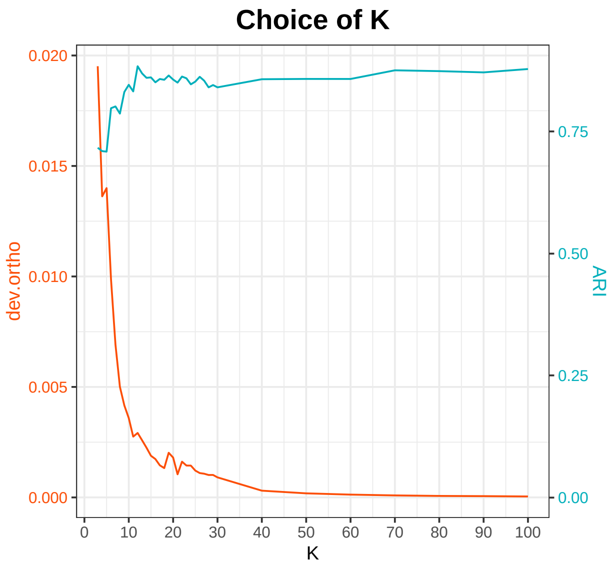

Figure S1: Comparison of *dev.ortho* and K-means ARI against low rank K on Zheng4 [Duò et al., 2018] dataset.

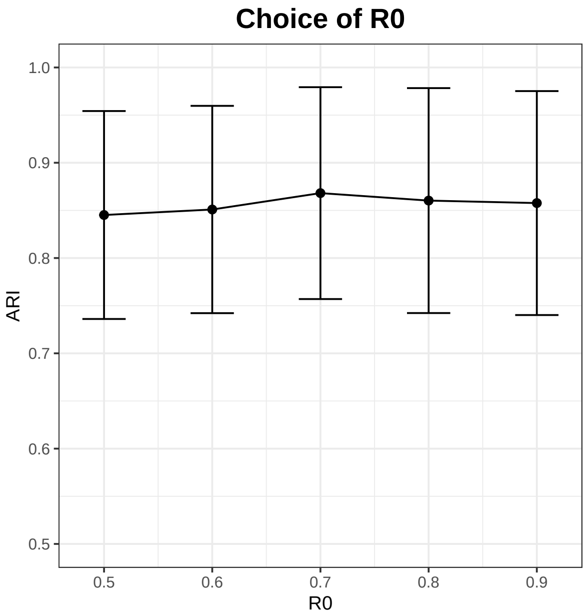

Figure S2: Comparison of K-means ARI against R_0, the threshold for correlations between score vectors and cell library sizes in scPNMF step II: basis selection. The mean ARI and the error bars are calculated across seven datasets (See Table S4).

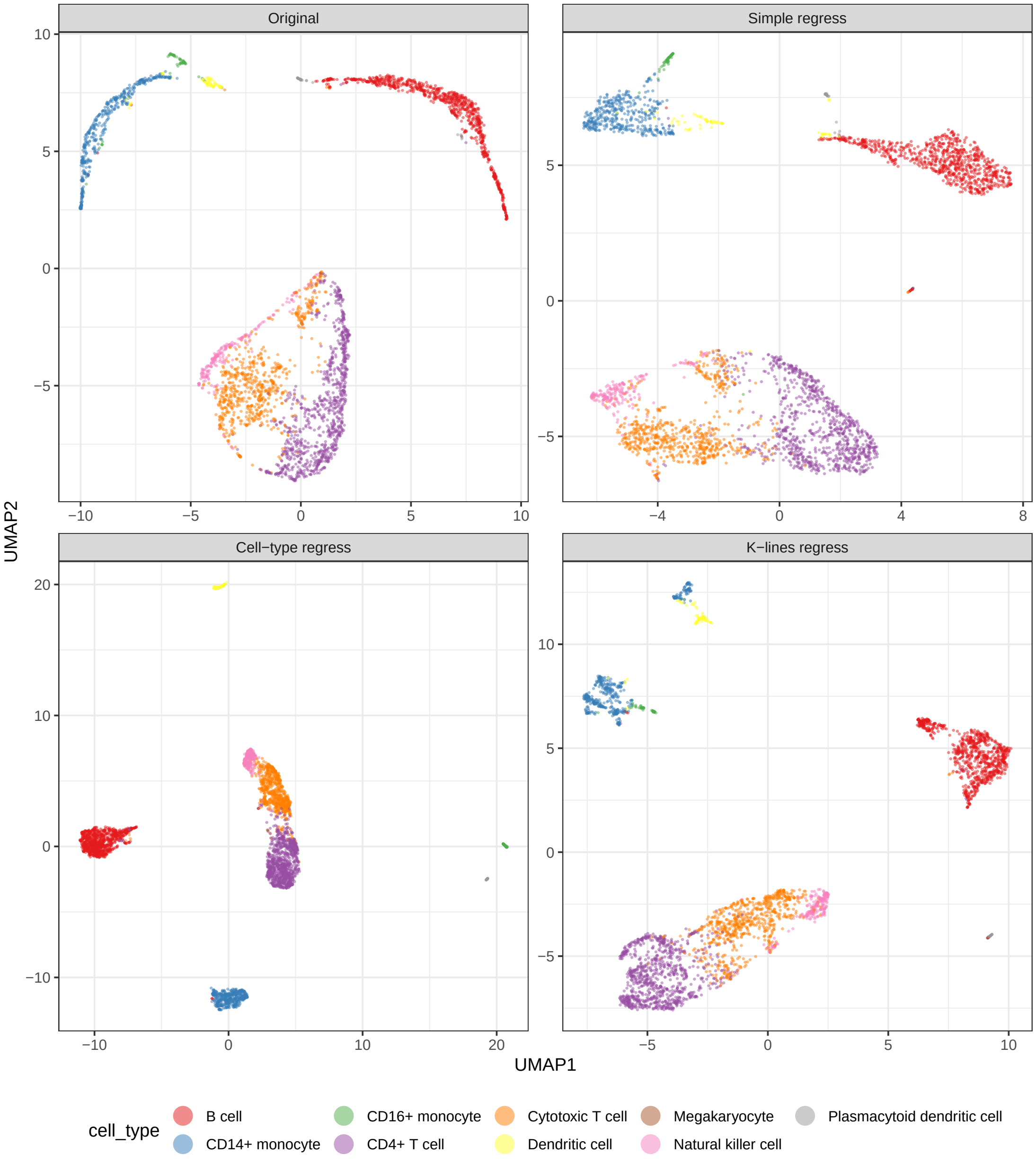

Figure S3: UMAP visualization of the cell score matrice $\mathbf{S}$ before correction and its corrected versions after regressing out cell library size in the PBMC10x dataset. Cell types are marked with colors. Original: the original score matrix $\mathbf{S}$ without correction; Simple regress: simply regressing out cell library size with all cells in one group, i.e., $N_k = 1$; Cell-type regress: regressing out cell library size within each cell type, i.e., $C_1, \ldots, C_{N_k}$ are cell types; K-lines regress: regressing out the library size within each K-lines cluster, i.e., $C_1, \ldots, C_{N_k}$ are cell clusters.

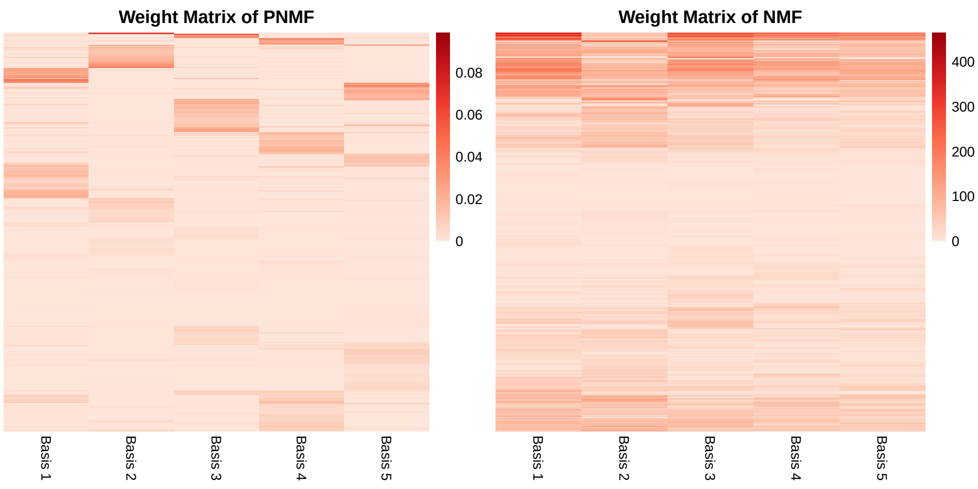

Figure S4: Weight matrices of PNMF and NMF. Rows are genes ordered by hierarchical clustering, and columns are bases.

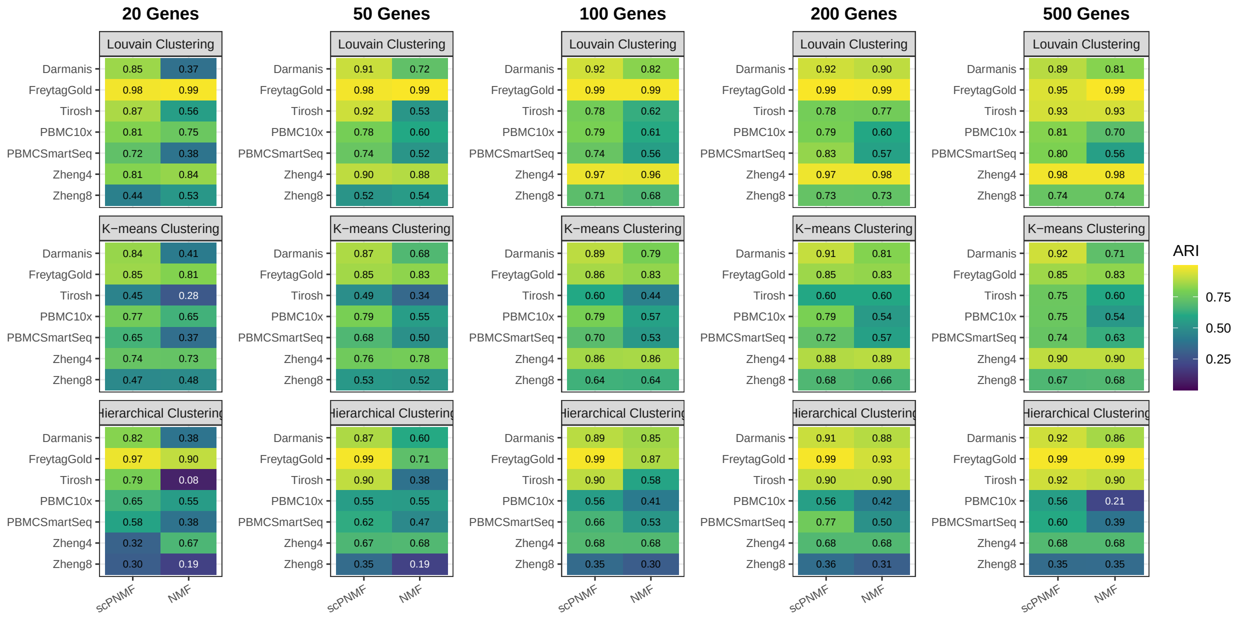

Figure S5: Benchmarking `scPNMF` and its variant, where PNMF is replaced by NMF, in selecting 20, 50, 100, 200, and 500 genes for cell clustering.

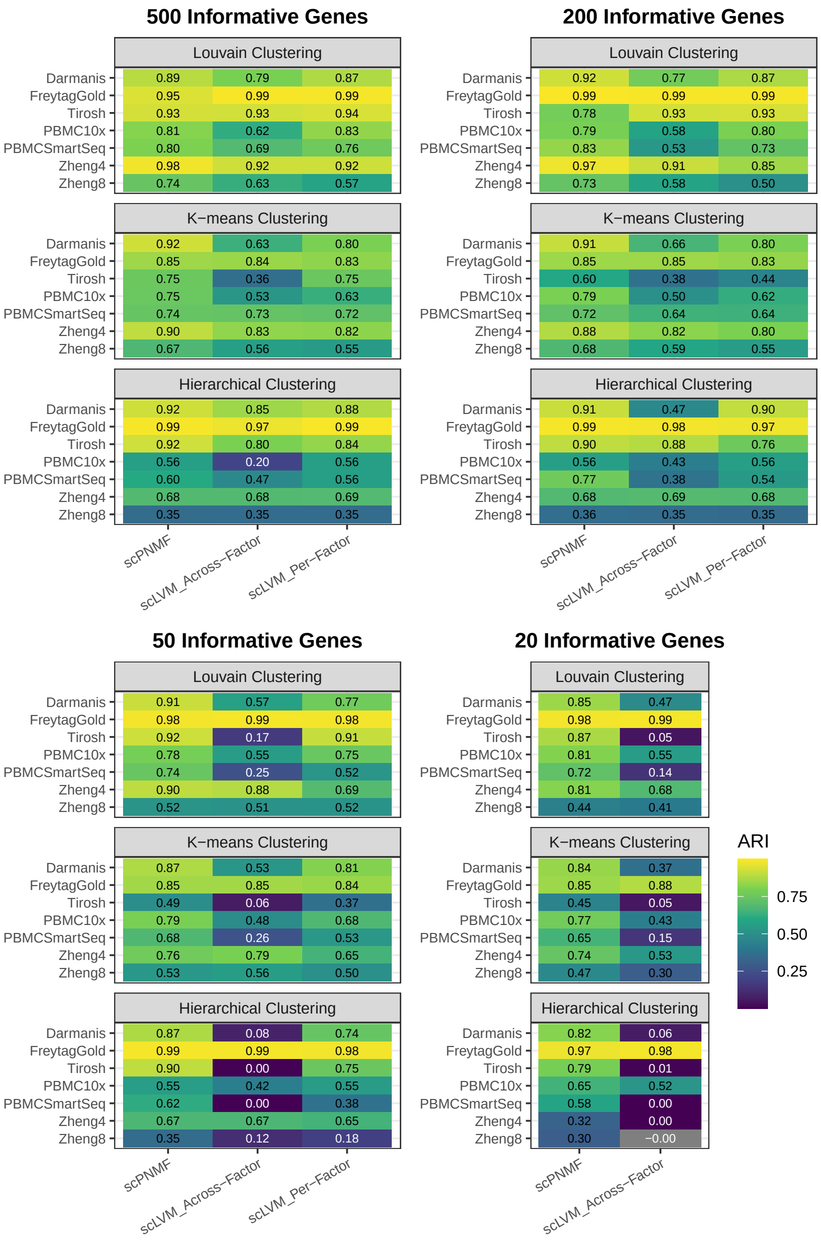

Figure S6: Benchmarking scPNMF and f-scLVM using 20, 50, 100, 200, 500 genes.

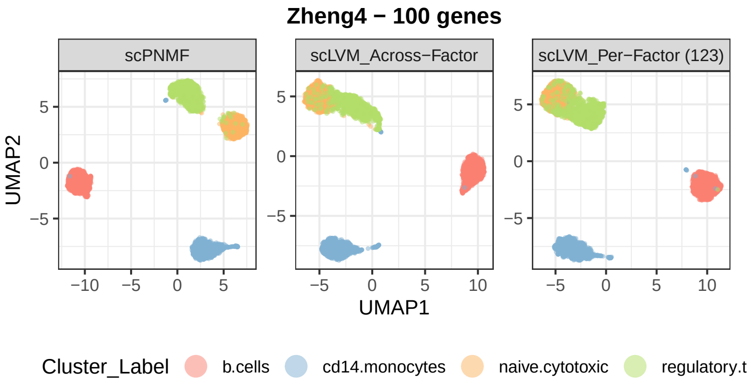

Figure S7: UMAP visualization of cells in the Zheng4 dataset based on 100 informative genes selected by scPNMF and f-scLVM.

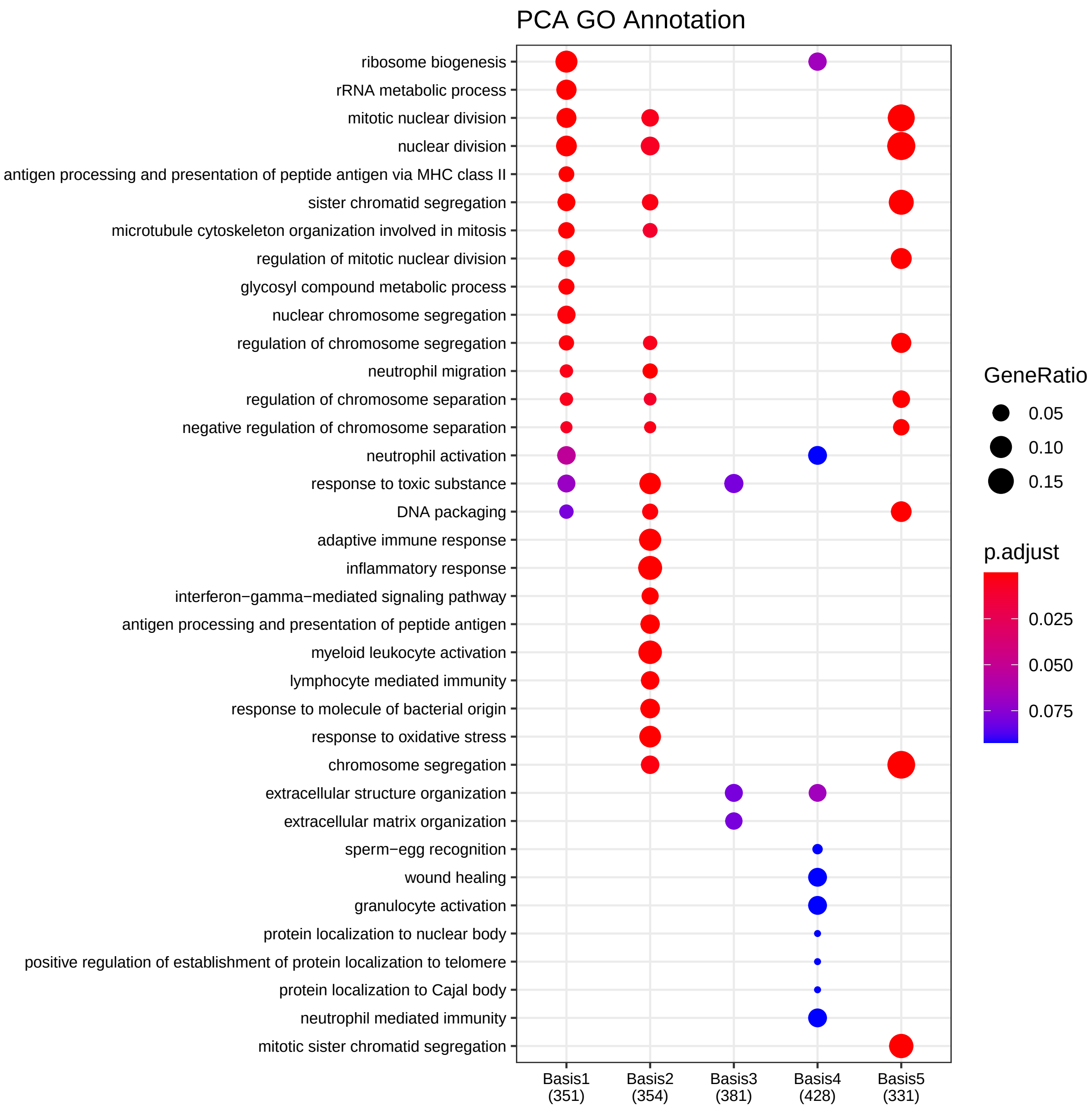

Figure S8: GO annotation on weight matrix of PCA. The enriched GO terms between basis are largely overlapped.

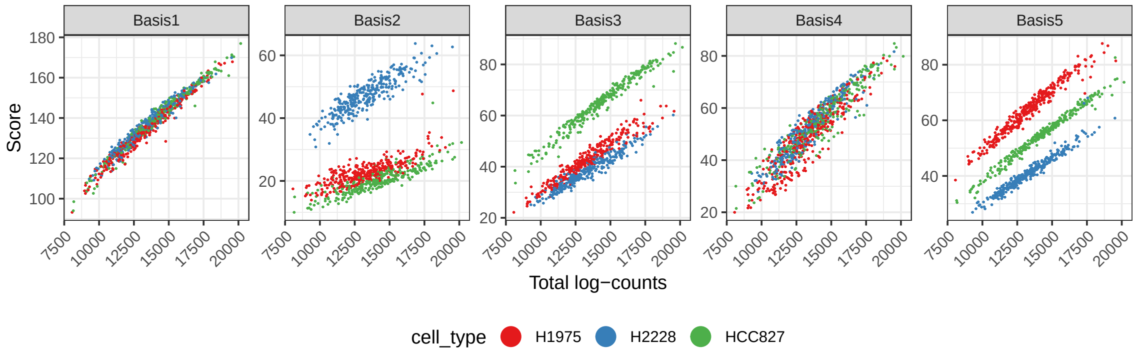

Figure S9: scPNMF scores versus total log-counts of FregGold dataset colored by cell types. Basis 2 distinguishes H2228 from the other two cell types and basis 3 distinguishes HCC827 from the other two cell types.

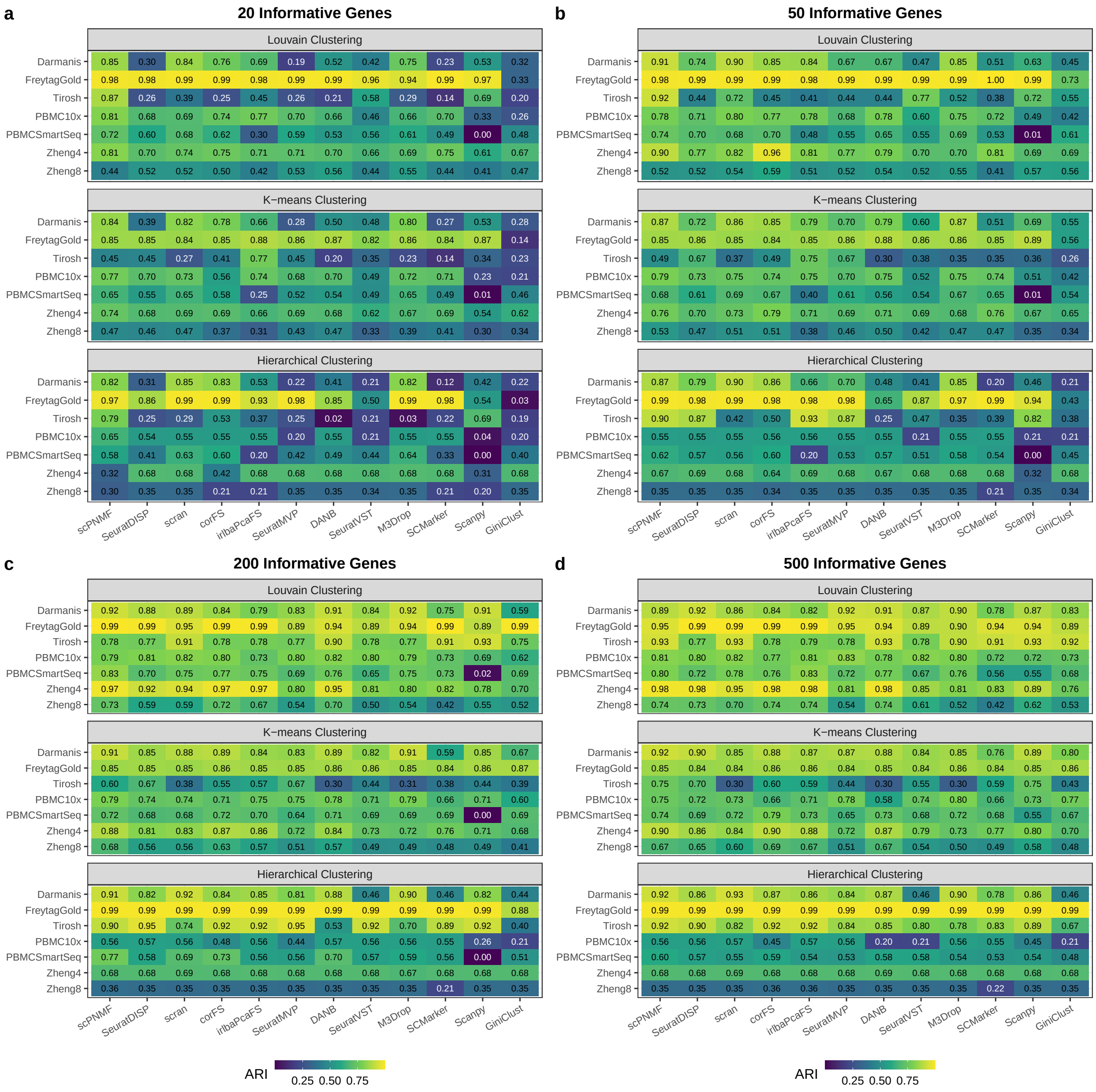

Figure S10: Benchmarking scPNMF and other informative gene selction methods using 20, 50, 200, 500 genes.

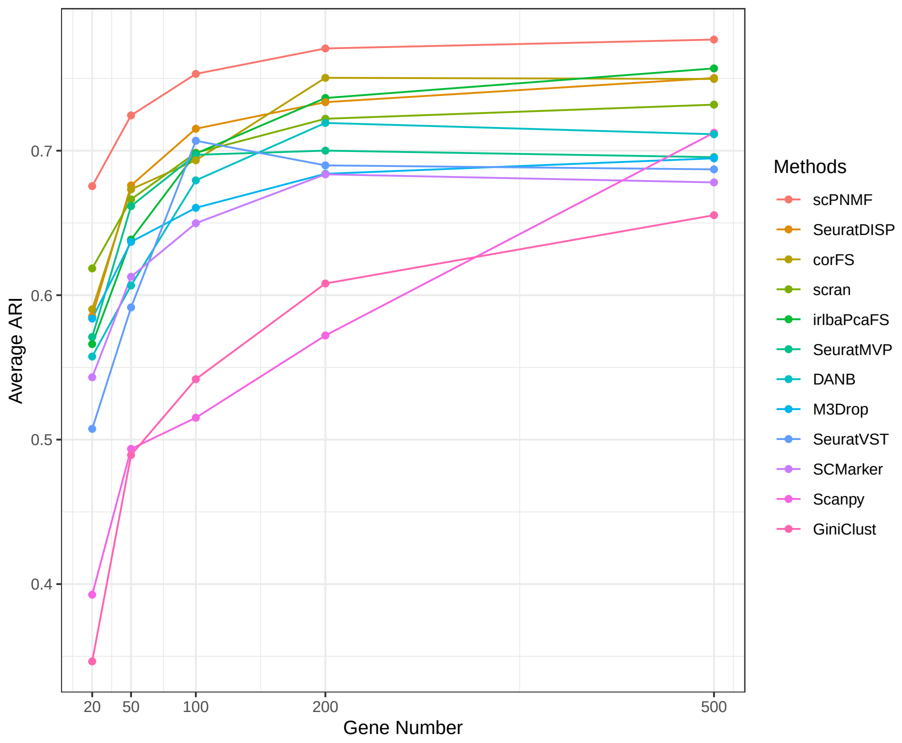

Figure S11: Comparison of overall average ARI of different methods versus gene numbers. The y-axis indicates the average ARI values across seven datasets and three clustering methods for each gene selection methods.

References

[Alquicira-Hernandez et al., 2019] Alquicira-Hernandez, J., Sathe, A., Ji, H. P., Nguyen, Q., and Powell, J. E. (2019). scpred: accurate supervised method for cell-type classification from single-cell rna-seq data. *Genome biology*, 20(1):1–17.

[Andrews and Hemberg, 2019] Andrews, T. S. and Hemberg, M. (2019). M3drop: dropout-based feature selection for scrnaseq. *Bioinformatics*, 35(16):2865–2867.

[Buettner et al., 2017] Buettner, F., Pratanwanich, N., McCarthy, D. J., Marioni, J. C., and Stegle, O. (2017). f-sclvm: scalable and versatile factor analysis for single-cell rna-seq. *Genome biology*, 18(1):1–13.

[Darmanis et al., 2015] Darmanis, S., Sloan, S. A., Zhang, Y., Enge, M., Caneda, C., Shuer, L. M., Gephart, M. G. H., Barres, B. A., and Quake, S. R. (2015). A survey of human brain transcriptome diversity at the single cell level. *Proceedings of the National Academy of Sciences*, 112(23):7285–7290.

[Ding et al., 2020] Ding, J., Adiconis, X., Simmons, S. K., Kowalczyk, M. S., Hession, C. C., Marjanovic, N. D., Hughes, T. K., Wadsworth, M. H., Burks, T., Nguyen, L. T., et al. (2020). Systematic comparison of single-cell and single-nucleus rna-sequencing methods. *Nature biotechnology*, pages 1–10.

[Duò et al., 2018] Duò, A., Robinson, M. D., and Soneson, C. (2018). A systematic performance evaluation of clustering methods for single-cell rna-seq data. *F1000Research*, 7.

[Freytag et al., 2018] Freytag, S., Tian, L., Lönnstedt, I., Ng, M., and Bahlo, M. (2018). Comparison of clustering tools in r for medium-sized 10x genomics single-cell rna-sequencing data. *F1000Research*, 7.

[Hafemeister and Satija, 2019] Hafemeister, C. and Satija, R. (2019). Normalization and variance stabilization of single-cell rna-seq data using regularized negative binomial regression. *Genome biology*, 20(1):1–15.

[Jiang et al., 2016] Jiang, L., Chen, H., Pinello, L., and Yuan, G.-C. (2016). Giniclust: detecting rare cell types from single-cell gene expression data with gini index. *Genome biology*, 17(1):144.

[Kim et al., 2020] Kim, T. H., Zhou, X., and Chen, M. (2020). Demystifying "drop-outs" in single-cell umi data. *Genome biology*, 21(1):1–19.

[Korsunsky et al., 2019] Korsunsky, I., Millard, N., Fan, J., Slowikowski, K., Zhang, F., Wei, K., Baglaenko, Y., Brenner, M., Loh, P.-r., and Raychaudhuri, S. (2019). Fast, sensitive and accurate integration of single-cell data with harmony. *Nature methods*, 16(12):1289–1296.

[Kuhn et al., 2008] Kuhn, M. et al. (2008). Building predictive models in r using the caret package. *J Stat Softw*, 28(5):1–26.

[Li et al., 2018] Li, J. J., Tong, X., and Bickel, P. J. (2018). Generalized pearson correlation squares for capturing mixtures of bivariate linear dependences. *arXiv preprint arXiv:1811.09965*.

[Lun et al., 2016] Lun, A. T., Bach, K., and Marioni, J. C. (2016). Pooling across cells to normalize single-cell rna sequencing data with many zero counts. *Genome biology*, 17(1):75.

[Monaco et al., 2019] Monaco, G., Lee, B., Xu, W., Mustafah, S., Hwang, Y. Y., Carre, C., Burdin, N., Visan, L., Ceccarelli, M., Poidinger, M., et al. (2019). Rna-seq signatures normalized by mrna abundance allow absolute deconvolution of human immune cell types. *Cell reports*, 26(6):1627–1640.

[Seurat, 2021] Seurat (2021). Analysis, visualization, and integration of spatial datasets with seurat.

[Stuart et al., 2019] Stuart, T., Butler, A., Hoffman, P., Hafemeister, C., Papalexi, E., III, W. M. M., Hao, Y., Stoeckius, M., Smibert, P., and Satija, R. (2019). Comprehensive integration of single-cell data. *Cell*, 177:1888–1902.

[Sun et al., 2020] Sun, T., Song, D., Li, W. V., and Li, J. J. (2020). scdesign2: an interpretable simulator that generates high-fidelity single-cell gene expression count data with gene correlations captured. *bioRxiv*.

[Tirosh et al., 2016] Tirosh, I., Izar, B., Prakadan, S. M., Wadsworth, M. H., Treacy, D., Trombetta, J. J., Rotem, A., Rodman, C., Lian, C., Murphy, G., et al. (2016). Dissecting the multicellular ecosystem of metastatic melanoma by single-cell rna-seq. *Science*, 352(6282):189–196.

[Wang et al., 2019] Wang, F., Liang, S., Kumar, T., Navin, N., and Chen, K. (2019). Scmarker: ab initio marker selection for single cell transcriptome profiling. *PLoS computational biology*, 15(10):e1007445.

[Wolf et al., 2018] Wolf, F. A., Angerer, P., and Theis, F. J. (2018). Scanpy: large-scale single-cell gene expression data analysis. *Genome biology*, 19(1):1–5.

[Yu et al., 2012] Yu, G., Wang, L.-G., Han, Y., and He, Q.-Y. (2012). clusterprofiler: an r package for comparing biological themes among gene clusters. *Omics: a journal of integrative biology*, 16(5):284–287.

[Zheng et al., 2017] Zheng, G. X., Terry, J. M., Belgrader, P., Ryvkin, P., Bent, Z. W., Wilson, R., Ziraldo, S. B., Wheeler, T. D., McDermott, G. P., Zhu, J., et al. (2017). Massively parallel digital transcriptional profiling of single cells. *Nature communications*, 8(1):1–12.
